# The relationship between different types of co-speech gestures and L2 speech performance

**DOI:** 10.3389/fpsyg.2022.941114

**Published:** 2022-08-16

**Authors:** Sai Ma, Guangsa Jin

**Affiliations:** ^1^Department of English Education, College of Foreign Languages, Capital Normal University, Beijing, China; ^2^Department of Linguistics, School of International Studies, University of International Business and Economics, Beijing, China

**Keywords:** gesture, L2 speech, meaning, form, discourse, representational, deictic, beat

## Abstract

Co-speech gestures are closely connected to speech, but little attention has been paid to the associations between gesture and L2 speech performance. This study explored the associations between four types of co-speech gestures (namely, iconics, metaphorics, deictics, and beats) and the meaning, form, and discourse dimensions of L2 speech performance. Gesture and speech data were collected by asking 61 lower-intermediate English learners whose first language is Chinese to retell a cartoon clip. Results showed that all the four types of co-speech gestures had positive associations with meaning and discourse L2 speech measures but no association with form-related speech measures, except the positive association between metaphorics and the percentage of error-free clauses. The findings suggest that co-speech gestures may have a tighter connection with meaning construction in producing L2 speech.

## Introduction

The widely accepted dimensions to evaluate L2 speech performance include complexity, accuracy, and fluency (CAF), among which complexity gauges to what degree speakers can use complex language forms, accuracy measures to what extent speakers produce errorless language forms, and fluency examines to what degree the speech is produced without unnecessary pauses (Skehan, 1998, 2009, 2014; Ellis and Barkhuizen, 2005). Since complexity and accuracy measures are associated with rule-based language knowledge, they represent the quality of language form; in contrast, fluency measures are linked to how quickly speakers convey their ideas, and thus they represent the quality of meaning (Skehan, 1998; Ellis and Barkhuizen, 2005).

L2 speech performance in terms of CAF has been found to interact with individual differences such as personality and anxiety (Oya et al., 2004), working memory (Mota, 2005), willingness to communicate (Nematizadeh and Wood, 2019), and sociocultural attitudes toward target language and culture (Sun, 2022), but few L2 speech studies have examined its association with co-speech gesturing, or specifically, the movements of the hand and arm during speech. Noteworthily, increasingly more researchers agree that speech and gesture are two different but closely related modalities to express thoughts (McNeill, 1992; Goldin-Meadow, 2003; Kendon, 2004), and their close associations have been empirically supported in terms of occurring time (Church et al., 2014), language development (Iverson and Goldin-Meadow, 2005; Vilà-Giménez et al., 2021), semantic content (Kita and Özyürek, 2003), pragmatic functions (Loehr, 2012), etc. Many relevant studies focused on native and bilingual speakers (Nicoladis et al., 2009; Smithson et al., 2011; Laurent et al., 2015), and the associations between speech and gesture use by L2 learners is under-researched.

This study complements the investigation of individual differences linked to L2 speech performance and the relationship between gesture and speech by exploring the associations between four types of co-speech gesture use and meaning, form and discourse aspects of L2 speech performance. In the remaining of this section, we first introduce the cognitive functions of co-speech gestures in speech production, and then review relevant studies on the associations between speech performance and co-speech gestures.

Co-speech gestures include iconics, metaphorics, deictics, and beats (McNeill, 1992). Iconics express concrete concepts by mimicking their size, shape, contour, etc.; metaphorics represent abstract concepts with concrete imageries created by movements of the hand and arm; deictics are pointing gestures that refer to an entity by the extending of the index finger, hand, or arm; and beats are biphasic up-down movements of the finger, hand, or arm (McNeill, 1992; Cartmill and Goldin-Meadow, 2016). Iconics and metaphorics are typical representational gestures that have a referential relationship with speech content.

Co-speech gestures, both on the whole and for each individual type, have been shown to be cognitively beneficial to speech. Working memory is a factor modulating and restricting L2 speech (Kormos, 2006; Weissheimer and Mota, 2009; Skehan, 2014), and co-speech gestures have been found to be able to reduce working memory load. For example, co-speech gestures can maintain mental imageries in memory and thus function to offload working memory burden during speech (Wesp et al., 2001). This function has also been supported by studies using the speech-memory dual task paradigm, in which participants memorized more items when they were allowed to gesture during the speech task than when they were prohibited to do so (Goldin-Meadow et al., 2001; Wagner et al., 2004; Ping and Goldin-Meadow, 2010; Cook et al., 2012), and the effect was especially obvious when participants' working memory capacity was low (Marstaller and Burianová, 2013).

Different types of co-speech gestures are also intimately linked to speech in terms of cognitive functions. Bearing a close relationship with speech content, representational gestures have been found to be beneficial cognitively for speech production. For instance, being embodied and three-dimensional, representational gestures can activate the image of concepts in mind and divide them into expressible units and thus help to scaffold speech content (Alibali et al., 2000; Kita and Davies, 2009; Chu et al., 2014). Meanwhile, using representational gestures can stimulate the linguistic representation of concepts in a cross-modal way, and thus helps speakers to retrieve words (Rauscher et al., 1996; Krauss et al., 2000; Frick-Horbury, 2002). Furthermore, speakers' working memory capacity has been found to be negatively associated with the use of representational gestures, suggesting that such gestures are strategies to compensate for the shortage of cognitive resources (Chu et al., 2014; Gillespie et al., 2014).

With either iconic gestures or iconic and metaphoric gestures together as the study object, the aforementioned studies on representational gestures failed to show the cognitive functions of metaphoric gestures. It has been proposed that both iconic and metaphoric gestures should be equally helpful in constructing concepts and speech (Kita et al., 2017). This proposal is reasonable in that, first, iconics and metaphorics have similar generating mechanisms, that is, both of them are based on the schematization of concepts, with iconics schematizing concrete concepts and metaphorics abstract ones (McNeill, 1992; Cienki and Müller, 2008; Chui, 2011; Burns et al., 2019); second, metaphors are conceptual in essence, so that people are very adept at producing and comprehending metaphorical gestures and speech, and usually employ metaphorical mappings unconsciously and effortlessly (Lakoff and Johnson, 1980; Cienki and Müller, 2008). Therefore, iconics and metaphorics may be similar in helping speech production. However, more empirical studies are in need.

Little research has examined the direct cognitive advantage of using deictics in speech, but deictic gesturing has been found to serve intrapersonal cognitive functions which are very likely to influence speech, such as lowering cognitive load (Ginns and King, 2021; Wang et al., 2022) and regulating attention (Delgado et al., 2009; Korbach et al., 2020). Empirical studies supporting such claims found that performing pointing gestures made learners do better in learning tasks (Hu et al., 2014, 2015; Agostinho et al., 2015; Ginns et al., 2016; Korbach et al., 2020; Ginns and King, 2021; Wang et al., 2022). Furthermore, tasks that require more cognitive effort, such as the verbal improvisation task, made participants generate more deictics than unchallenging tasks, such as the ordinary verbal task, indicating that deictic gesturing is a strategy to lower cognitive load (Lewis et al., 2015). It is also worth mentioning that in studies that have shown gestures' function of lowering cognitive load using the speech-memory dual task paradigm, a large percentage of gestures used in the gesture-allowed condition were deictic ones (Goldin-Meadow et al., 2001; Wagner et al., 2004; Ping and Goldin-Meadow, 2010; Cook et al., 2012).

Contrary to representational and deictic gestures, beats have little relationship with speech content but are associated with discourse features of the accompanying speech (McNeill, 1992; Dimitrova et al., 2016; Shattuck-Hufnagel and Ren, 2018). Beat gesturing has been found to be beneficial to solving tip-of-the-tongue problems (Ravizza, 2003), retrieving low-frequency words (Lucero et al., 2014), encoding and recalling foreign words (Morett, 2014), and enhancing children's narrative structure and fluency while narrating stories (Vilà-Giménez and Prieto, 2020). These studies show that beats are helpful cognitively in accessing lexicons, structuring narration, and improving fluency.

We next review literature on the associations between co-speech gestures and speech performance. Gesturers tend to do better than non-gesturers in fluency, lexical richness, and speech length for both native and bilingual speakers. For example, gesture restriction worsened fluency measures for English native speakers (Rauscher et al., 1996) and reduced the number of word types, word tokens, and scenes for bilinguals (Laurent and Nicoladis, 2015); in addition, gesturers spoke faster and produced more word tokens than non-gesturers for bilinguals (Smithson et al., 2011).

Individual types of co-speech gestures are associated with speech performance in different ways. Iconics, which have drawn the most scholarly attention, have been observed to relate to speech performance either positively or neutrally. Studies have shown that the iconic gesture rate was positively correlated with the number of word types for native French speakers (Nicoladis et al., 2009), with the number of word tokens for native English speakers (Smithson et al., 2011), and with the number of scenes (Laurent et al., 2015) and the length of utterances (Nicoladis et al., 1999) for bilingual kids; but it had no relationship with the number of word tokens for Chinese-English or French-English bilinguals (Smithson et al., 2011), with the number of word types for English native speakers (Nicoladis et al., 2009) or English-French bilinguals (Nicoladis et al., 2009; Laurent et al., 2015), or with the speech rate for either native or bilingual speakers (Smithson et al., 2011). In addition, the number of word tokens could predict the iconic gesture rate for Spanish, English, and French native speakers (Nicoladis et al., 2016), but the speech rate could not predict it for bilinguals (Aziz and Nicoladis, 2019).

Most of the above studies invited either native speakers or highly proficient L2 speakers to complete a cartoon-retelling task, which might be a reason for the mixed results. Both native and highly proficient speakers are less likely to find speech tasks challenging, in which case their available working memory resources are adequate for the task and their gestures are mainly a reflection of their speaking styles (Nagpal et al., 2011). On the other hand, less proficient L2 speakers' gesture use has closer association with speech since they need strategies like gesturing to compensate for the shortage of working memory resources during speaking (Nicoladis et al., 2007; Aziz and Nicoladis, 2019). For example, Nicoladis et al. (2007) found that the positive correlation between the iconic rate and the number of scenes described only held for intermediate Chinese English learners but not for native Chinese speakers. Unfortunately, little attention has been paid to the relationship between gesture and speech produced by L2 learners except for the study conducted by Nicoladis et al. (2007) and Ma et al. (2021). Ma et al. (2021) found that, for both concrete and abstract speech content, lower-intermediate L2 learners' representational gesture use was positively associated with speech fluency measures and speech length.

Research specifically investigating the relationship between metaphoric gestures and speech performance has been rare. It is necessary to verify the proposal that metaphorics should bear similar cognitive functions to iconics in speech production (Kita et al., 2017) through exploring the association between metaphoric gesturing and speech performance.

The relationship between using deictics and speech performance has been scarcely examined. Nicoladis (2002) found that the number of deictics was positively associated with the number of utterances for French-English bilingual preschoolers. Several studies concluded that the associations of speech with iconics and beats differ from its association with deictics in that the use of iconics and beats develops with speech proficiency whereas deictic gesturing compensates for weak language proficiency (Nicoladis et al., 1999; Mayberry and Nicoladis, 2000; Nicoladis, 2002; Gan and Davison, 2011; Lin, 2019). This conclusion is supported by findings that iconic and beat gesture use bore stronger correlations with speech measures than deictic gesture use did (Nicoladis et al., 1999; Mayberry and Nicoladis, 2000), that bilinguals used more iconics instead of deictics in their dominant language (Nicoladis, 2002), that L2 learners used more deictics rather than iconics in L2 discussion (Gan and Davison, 2011), and that L2 learners with higher proficiency produced more iconics and beats but fewer deictics than less proficient L2 speakers (Lin, 2019).

Beats have been found to be associated positively with some aspects of speech performance. The use of beats was correlated positively with the length of utterances, the number of word types, and the number of scenes for bilingual kids (Nicoladis et al., 1999; Laurent et al., 2015). Furthermore, when explainers explained foreign words to learners, both explainers' and learners' beat gesture use predicted the number of word tokens they produced (Morett, 2014). Beat gesturing has been observed to co-occur with the use of connectives in the discourse (McNeill, 1992; Levy and McNeill, 2013), and as speakers' ability to establish discourse cohesion increased, their use of beats also went up (Alamillo et al., 2013; Colletta et al., 2015). It can be seen that beats are associated positively with the length of speech units, speech length, and lexical richness, but the quantitative association between beat gesturing and discourse-associated speech measures requires further investigation.

Previous research has provided valuable findings in the associations between co-speech gesturing and speech performance, but there are still important aspects to be examined. First, a large percentage of relevant studies focused on representational gestures, leaving the relationship between individual types of co-speech gestures and speech performance insufficiently studied. Second, most studies on gesture and speech did not situate themselves in the framework of L2 acquisition, so they failed to fully consider how L2 speech performance is measured in the area of applied linguistics and thus could not give a full picture of the associations between gesturing and L2 speech performance. Third, the literature that has explored the associations between gesture use and speech performance largely focused on native speakers and highly proficient speakers, whose gesture use was probably associated more with personal speaking styles than with the speech production process, and thus such studies have yielded mixed results. Less attention has been paid to L2 learners whose gesture use is more likely to facilitate speech, and research on this topic also has pedagogical implications.

In view of the above research gaps, this study aims to explore the associations between different types of co-speech gestures and L2 speech performance. All of the four types of co-speech gestures were considered, and the meaning, form, and discourse aspects of L2 speech performance were measured (see Section “Speech transcription and coding” for details). The specific research question is: To what extent is each type of co-speech gestures associated with the meaning, form, and discourse aspects of L2 speech performance?

## Methodology

### Participants

Participants were 61 lower-intermediate level English learners in a Chinese university. They were recruited from a spoken English course for first year non-English major undergraduates taught by the second author. The speech task that provided speech and gesture data was one of the class activities. Students who agreed to have their data studied were offered a coupon for cake. All students agreedto participate.

Our participants included 24 male and 37 female students aged from 17 to 19 (*M* = 17.98; *SD* = 0.46). They were all Han Chinese with Chinese as their mother tongue, English the second language and no third language. They started to learn English from primary school and had been enrolled in the undergraduate program for about 8 weeks when the study took place. At the time of enrollment, they were categorized as intermediate-level English learners based on an English proficiency test provided by the i-Test system designed by Foreign Language Teaching and Research Press. The test, with a written component and a spoken component, examines learners' comprehensive English abilities. Intermediate English learners in this university were required to study English for two semesters before taking the nationwide College English Test, band 4 (CET-4). CET-4 matches the fifth level of China's Standards of English Language Ability scale (Wang, 2018), which corresponds to IELTS 5.5 and the lower B2 level of CEFR. Therefore, the English proficiency level of our participants was regarded as lower-intermediate.

### Instrument

A cartoon-retelling task was used to elicit speech and gesture data. The task used a 30-s cartoon clip of *Tom and Jerry*, in which Tom played with Jerry by running after him, catching him, and letting him go time after time, and Jerry was trying to escape from Tom with all his might. The cartoon is rich in movements, actions, and trajectories, which is conducive to gesturing. The instructions to the participants were as follows: *You are going to see a clip of a cartoon. After watching it, please describe what happened in the clip*.

### Procedures

Participants were told that this classroom activity was also intended as the data source of a study on how to improve L2 learners' spoken English. It was made clear that students who agreed to have their data used would be rewarded with a coupon for cake, and students who chose not to participate would not be affected in any way. After filling the general information sheet and consent form, students were led to a nearby classroom one by one, where the experimenter stated the task requirements and showed each of them the video clip. Participants were asked to do the retelling immediately after watching the clip, and there was no time limit for the retelling. During this process, participants stood opposite the experimenter so that their gestures could be captured clearly, and they were required to keep their hands empty. They were allowed to ask the experimenter to clarify the task requirement. A video camera was used to record participants' performance. A quick post-experiment survey showed that no one had been aware that the study intended to elicit gesture data. Afterwards, the real purpose of the study was revealed to the participants, and they could choose to withdraw from the study within 3 months from the experiment date, and none did so.

### Speech transcription and coding

Students' speeches were transcribed verbatim and analyzed. In choosing speech performance measures for this study, we referred to the widely accepted complexity, accuracy, and fluency dimensions in evaluating L2 speech performance and adopted the relevant notion that L2 speech performance includes meaning and form associated aspects. In addition, we also considered the aspects of speech performance that have been reported to be empirically or theoretically associated with gesturing. Based on relevant literature, co-speech gesturing is related to speech length, lexical richness, speaking fluency, the length of certain speech units, and discourse cohesion. Speech length pertains to the productivity in communicating messages, lexical richness reflects the diversity of words used to express thoughts, and speaking fluency represents how quickly the speaker intends to covey ideas; thus, they were all treated as meaning-associated speech measures in this study. The length of certain speech units reflects how complex a clause or utterance is, and thus was taken as a measure related to form. Discourse cohesion reflects speakers' ability to produce text within which there are associated meaning units and establish the underlying meaning structure on the discourse level (Halliday and Hasan, 1976), and it was considered a separate dimension of L2 speech performance, namely, the discourse dimension.

This study employed three meaning-associated speech measures, including the number of word tokens to measure speech length, the root type/token ratio (RTTR) to measure lexical richness, and the speech rate to measure fluency. Although little research has shown significant association between form-associated speech measures and gesturing, we still checked this possibility since gesturing is believed to have the function of lowering working memory load in general. Three widely used form-associated measures were chosen, including the percentage of subordination to measure syntactic complexity (a complexity measure), the mean length of clauses to measure clause complexity (a complexity measure), and the percentage of error-free clauses to measure speech correctness (an accuracy measure). We employed the number of connectives per clause to measure discourse cohesion. In sum, seven speech measures were employed to represent the meaning, form, and discourse dimensions of L2 speech performance.

Table 1 shows how meaning, form, and discourse-associated speech measures were calculated. In calculating form-associated speech measures, the unit adopted is the Analysis of Speech Unit (Foster et al., 2000, p. 365–366), which is defined as “an independent clause or an independent sub-clausal unit together with any associated subordinate clause(s).” Clauses consisted of the Analysis of Speech Units and subordinate clauses.

**Table 1:** The calculations of speech measures.

**Meaning-associated speech measures**
Number of word tokens: The total number of word tokens
RTTR: The number of word types divided by the square root of the number of word tokens
Speech rate: The number of syllables divided by sample time (in min)
**Form-associated speech measures**
Percentage of subordination: The number of subordinate clauses divided by the number of clauses
Mean length of clauses: The number of word tokens divided by the number of clauses
Percentage of error-free clauses: The number of error-free clauses divided by the number of clauses
**Discourse-associated speech measures**
Number of connectives per clause: The total number of connectives including coordinating and subordinating conjunctions, connective adverbs, and other connective expressions, divided by the number of clauses

To calculate the above meaning, form, and discourse-associated speech measures, we first removed repair disfluencies from the verbatim transcription, such as repetitions, self-corrections, hesitations, and speech irrelevant to the cartoon-retelling; we then counted speech data in terms of the number of word tokens, word types, syllables, connectives, the Analysis of Speech Units, subordinate clauses, clauses, and error-free clauses, and we also annotated sample duration. For annotations that could not be generated automatically, i.e., connectives, the Analysis of Speech Units, subordinate clauses, and error-free clauses, the first author did the coding first and a research assistant coded 20% (13 participants) of the data. The agreement was 100% (*N* = 117) for connectives, 92.25% (*N* = 142) for the number of the Analysis of Speech Units, 97.56% (*N* = 41) for subordinate clauses, and 100% (*N* = 44) for error-free clauses.

### Gesture coding

We followed McNeill (1992) to categorize co-speech gestures into iconics, metaphorics, deictics, and beats. Gestures were coded in ELAN (Lausberg and Sloetjes, 2009). Gesture types were annotated based on both the gesture form and the accompanying speech content. Participants often used iconics to mimic the moving route and actions of Tom and Jerry; metaphorics were employed to accompany abstract speech content such as process, time, and emotion; deictics were adopted when participants referred to the protagonists of the cartoon, Tom and Jerry, as well as to their body parts associated with movements, such as Jerry's tail and Tom's mouth; beats were the most frequently used type of gestures for all participants.

**Figure 1:**
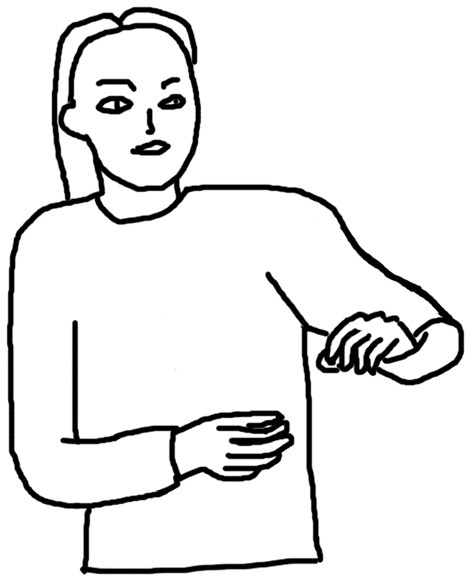
An example of an iconic gesture.

**Figure 2:**
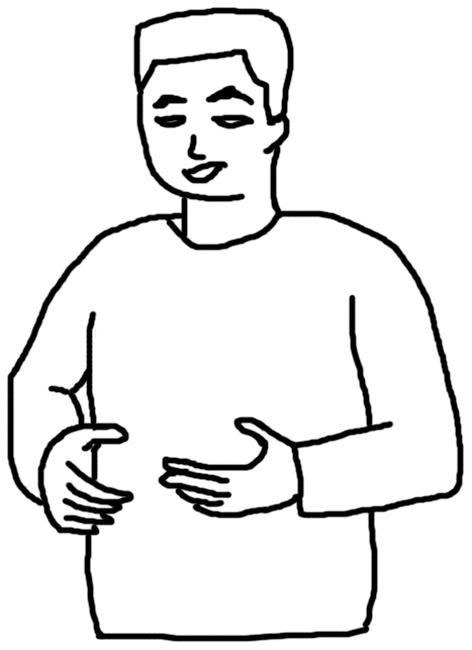
An example of a metaphoric gesture.

**Figure 3:**
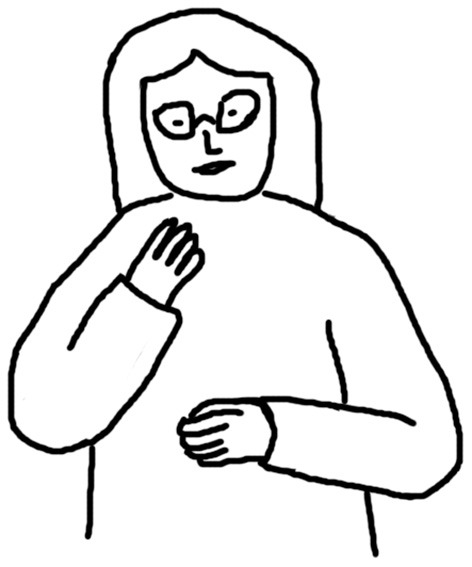
An example of a deictic gesture.

**Figure 4:**
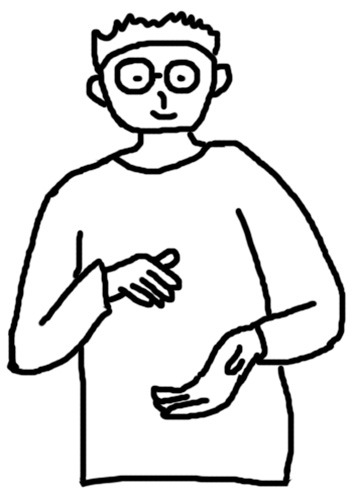
An example of a beat gesture.

Figures 1–4 illustrate examples of the iconic, metaphoric, deictic, and beat gestures respectively. The gesture in Figure 1 was made when the participant said *the cat use its fingers to kind of catch and hold the tail of the mouse*. Her right hand was kept still; meanwhile, her left elbow lifted, and her left thumb and forefinger reached out and touched each other as if she was pinching something. Obviously, she was mimicking the action of the cat, and thus this gesture was coded as an iconic gesture. The participant in Figure 2 said *and the cat is quite enjoying this process* when he made the gesture. His two hands were in front of him and near each other and he rotated his elbows so that his hands were circling like a wheel. This gesture was coded as a metaphoric one since it mimicked the abstract concept “process” with the circling of both hands. Figure 3 shows a deictic gesture when the participant said *and then he open his mouth and jerry run straight to his mouth*. Her left hand was in a relaxing position, and she pointed her right hand toward her mouth. Figure 4 illustrates the use of beats. The participant kept his left hand still and moved his right hand up and down repeatedly when he said *well several times Jerry went into the Tom's mouth*.

All gesture data were annotated by the first author, and then 20% of the data (data of 13 participants) were coded by a research assistant unaware of the research purpose. The agreement was 71.43% (*N* = 42) for iconics, 84.21% (*N* = 5) for metaphorics, 100% (*N* = 18) for deictics, and 88% (*N* = 111) for beats. We used the gesture rate as the gesture measure, which was calculated by dividing the number of each type of gestures by the number of word tokens and multiplying the result with 100.

### Statistical analysis

We used both difference analysis and correlation analysis to show the relationship between co-speech gesturing and L2 speech performance. ANOVA was used to compare L2 speech measures for gesturers and non-gesturers for each co-speech gesture type. Results of the Mann–Whitney *U*-test were reported for cases involving metaphorics as there was considerable difference in the number of gesturers and non-gesturers (see **Table 4**). Welch correction was adopted for cases where Levene's test reached significance. Since the gesture frequency data were not normally distributed, Spearman's rho was adopted to explore the correlational relationship between different types of co-speech gesture rate and speech measures.

## Results

We first show descriptive statistics of the speech and gesture data, then display the results of difference analysis and correlation analysis, and finally answer the research question by summarizing the calculation results.

### Descriptive statistics of speech and gesture data

Table 2 shows the means, standard deviations, and ranges of the data used in the calculations of speech measures and the descriptive statistics of the speech measures.

**Table 2:** Descriptive statistics of data used in speech measure calculations and those of speech measures.

	***Mean***	***SD***	***Range***
**Descriptive statistics of data used in speech measure calculations**			
Number of syllables	73.69	48.11	20–241
Number of the analysis of speech units	8.67	5.57	2–28
Number of subordinations	2.64	2.04	0–9
Number of error-free clauses	3.77	3.18	0–14
Sample duration (in s)	62.46	27.21	17.6–125.74
**Descriptive statistics of speech measures**			
**Meaning-associated speech measures**			
Number of word tokens	61.20	38.91	19–180
RTTR	4.056	0.758	2.524–5.859
Speech rate	71.55	28.9	13.67–138.40
**Form-associated speech measures**			
Percentage of subordination	0.23	0.16	0–0.71
Mean length of clauses	5.43	1.00	3.14–8.25
Percentage of error-free clauses	0.33	0.23	0–1
**Discourse-associated speech measures**			
Number of connectives per clause	0.795	0.31	0.182–1.571

Table 3 illustrates the descriptive statistics of both the token and frequency of each type of gestures. It can be seen that the most frequently used gesture type was the beat, followed by the iconic, and then by the deictic. Metaphorics were used least, which was probably due to the largely concrete content involved in the cartoon clip. Table 4 shows the number of participants that used and did not use each type of gestures and the descriptive statistics of the gesture rate for gesturers. The descriptive statistics of speech data for gesturers and non-gesturers of each type of gesture are displayed in Tables 5–8. Please note that non-gesturers of a certain type of gesture may have used other types of gestures (e.g., a non-gesturer of iconics may be a gesturer of beats).

**Table 3:** Descriptive statistics of gesture tokens and frequency.

	**Token**			**Frequency**		
	***Mean***	***SD***	***Range***	***Mean***	***SD***	***Range***
Iconics	3.21	4.75	0–24	4.31	5.45	0–21.43
Metaphorics	0.56	1.42	0–8	0.58	1.36	0–5.56
Deictics	1.38	2.46	0–12	2.13	3.40	0–12.90
Beats	7.07	10.08	0–41	9.78	11.94	0–45.71

**Table 4:** Number of gesturers and non-gesturers of four types of gestures.

	**Number of gesturers (*Mean*, *SD*, and *Range* of gesturers' gesture rate)**	**Number of non-gesturers**
Iconics	33 (*M* = 7.966; *SD* = 5.067; *Range* = 0.654–21.429)	28
Metaphorics	13 (*M* = 2.744; *SD* = 1.698; *Range* = 0.654–5.556)	48
Deictics	27 (*M* = 4.809; *SD* = 3.654; *Range* = 1.053–12.903)	34
Beats	33 (*M* = 18.081; *SD* = 10.599; *Range* = 3.061–45.714)	28

**Table 5:** Descriptive statistics of speech measures for gesturers and non-gesturers of iconics.

	**Gesturers (iconics)**			**Non-gesturers (iconics)**		
	***Mean***	***SD***	***Range***	***Mean***	***SD***	***Range***
**Meaning-associated speech measures**						
Number of word tokens	78.424	40.894	19–180	40.893	24.33	19–103
RTTR	4.409	0.742	2.524–5.859	3.639	0.539	2.772–4.704
Speech rate	83.875	26.136	29.138–138.382	57.018	25.329	13.67–111.111
**Form-associated speech measures**						
Percentage of subordination	0.202	0.12	0–0.474	0.272	0.188	0–0.714
Mean length of clauses	5.488	0.809	3.923–7.176	5.352	1.196	3.143–8.25
Percentage of error-free clauses	0.312	0.191	0–0.714	0.359	0.27	0–1
**Discourse-associated speech measures**						
Number of connectives/clause	0.913	0.267	0.421–1.571	0.655	0.302	0.182–1.389

**Table 6:** Descriptive statistics of speech measures for gesturers and non-gesturers of metaphorics.

	**Gesturers (metaphorics)**			**Non-gesturers (metaphorics)**		
	***Mean***	***SD***	***Range***	***Mean***	***SD***	***Range***
**Meaning-associated speech measures**						
Number of word tokens	99.615	42.9	45–180	50.792	30.723	19–147
RTTR	4.767	0.653	3.709–5.859	3.863	0.668	2.524–5.379
Speech rate	100.408	25.63	56.621–138.382	63.731	24.609	13.67–111.111
**Form-associated speech measures**						
Percentage of subordination	0.201	0.107	0–0.4	0.243	0.168	0–0.714
Mean length of clauses	5.645	0.705	4.357–7.176	5.366	1.063	3.143–8.25
Percentage of error-free clauses	0.429	0.109	0.235–0.625	0.308	0.247	0–1
**Discourse-associated speech measures**						
Number of connectives/clause	0.923	0.243	0.571–1.29	0.76	0.319	0.182–1.571

**Table 7:** Descriptive statistics of speech measures for gesturers and non-gesturers of deictics.

	**Gesturers (deictics)**			**Non-gesturers (deictics)**		
	***Mean***	***SD***	***Range***	***Mean***	***SD***	***Range***
**Meaning-associated speech measures**						
Number of word tokens	74.074	40.539	19–180	50.971	34.847	19–153
RTTR	4.339	0.841	2.524–5.859	3.831	0.607	2.772–5.114
Speech rate	82.501	27.358	38.182–138.382	62.849	27.433	13.67–116.505
**Form-associated speech measures**						
Percentage of subordination	0.202	0.136	0–0.5	0.26	0.17	0–0.714
Mean length of clauses	5.545	0.717	3.923–7.176	5.331	1.178	3.143–8.25
Percentage of error-free clauses	0.316	0.202	0–0.714	0.348	0.252	0–1
**Discourse-associated speech measures**						
Number of connectives/clause	0.922	0.3	0.421–1.571	0.694	0.282	0.182–1.308

**Table 8:** Descriptive statistics of speech measures for gesturers and non-gesturers of beats.

	**Gesturers (beats)**			**Non-gesturers (beats)**		
	***Mean***	***SD***	***Range***	***Mean***	***SD***	***Range***
**Meaning-associated speech measures**						
Number of word tokens	73.212	40.44	19–180	47.036	32.274	19–147
RTTR	4.272	0.802	2.524–5.859	3.801	0.625	2.772–5.114
Speech rate	81.275	28.124	13.67–138.382	60.083	25.816	17.817–111.111
**Form-associated speech measures**						
Percentage of subordination	0.209	0.121	0–0.474	0.264	0.19	0–0.714
Mean length of clauses	5.355	0.877	3.143–7.176	5.508	1.137	4.182–8.25
Percentage of error-free clauses	0.306	0.181	0–0.625	0.367	0.276	0–1
**Discourse-associated speech measures**						
Number of connectives/clause	0.889	0.287	0.286–1.571	0.684	0.303	0.182–1.389

### Results of difference analysis: The presence and absence of co-speech gestures and L2 speech performance

Table 9 shows whether speech measures were different due to the presence and absence of individual types of co-speech gestures. Specifically, participants who used iconics produced speeches that were better in the number of word tokens (*F* = 19.614, *p* < 0.001), RTTR (*F* = 20.826, *p* < 0.001), the speech rate (*F* = 16.453, *p* < 0.001), and the number of connectives per clause (*F* = 12.57, *p* < 0.001), whereas using iconics did not make much difference in the percentage of subordination, the mean length of clauses, and the percentage of error-free clauses.

**Table 9:** The contrast of speech measures produced by gesturers and non-gesturers of four types of co-speech gestures.

	**Iconics**	**Metaphorics**	**Deictics**	**Beats**
***F*** **(**ω**) for iconics, deictics, beats;** ***U*** **(*****r*****) for metaphorics**				
**Meaning-associated speech measures**				
Number of word tokens	19.614*** (0.468)	530*** (0.492)	5.724* (0.268)	7.611** (0.313)
RTTR	20.826*** (0.495)	517*** (0.462)	7.517** (0.311)	6.354* (0.285)
Speech rate	16.453*** (0.449)	527*** (0.485)	7.742** (0.316)	9.268** (0.345)
**Form-associated speech measures**				
Percentage of subordination	3.087 (0.182)	256 (−0.127)	2.061 (0.13)	1.703 (0.114)
Mean length of clauses	0.275 (0)	387 (0.169)	0.765 (0)	0.351 (0)
Percentage of error-free clauses	0.641 (0)	448.5* (0.308)	0.282 (0)	1.056 (0.032)
**Discourse-associated speech measures**				
Number of connectives per clause	12.57*** (0.399)	412 (0.226)	9.314** (0.346)	7.345** (0.307)

* p < 0.05,

** p < 0.01,

*** p < 0.001.

Similar to the case of iconics, participants who used metaphorics produced speech with more word tokens (*U* = 530, *p* < 0.001), higher RTTR (*U* = 517, *p* < 0.001), and faster speed (*U* = 527, *p* < 0.001) than participants who did not. The number of connectives per clause also trended toward significance (*U* = 412, *p* = 0.077), and the percentage of subordination and the length of clauses did not reach significance. Compared with participants who did not use metaphorics, participants who used them produced a higher percentage of error-free clauses (*U* = 448.5, *p* = 0.016), which is a form-associated measure.

The cases for deictic and beat gesturing resembled those of iconic gesturing, only with weaker effects. Deictic gesturers produced a significantly larger number of word tokens (*F* = 5.724, *p* = 0.02), higher RTTR (*F* = 7.517, *p* = 0.008), higher speech rate (*F* = 7.742, *p* = 0.007), and more connectives per clause (*F* = 9.314, *p* = 0.003) than non-gesturers of deictics, but none of the three form-associated measures showed any significant difference. Likewise, beat gesturers performed better than non-gesturers of beats in the number of word tokens (*F* = 7.611, *p* = 0.008), RTTR (*F* = 6.354, *p* = 0.014), the speech rate (*F* = 9.268, *p* = 0.003), and the number of connectives per clause (*F* = 7.345, *p* = 0.009), but speeches produced by beat gesturers and non-gesturers did not differ in any of the three form-associated speech measures.

### Results of correlation analysis: The correlations between co-speech gestures and L2 speech performance

Table 10 shows the correlational relationships between different types of co-speech gesture rate and the speech measures associated with meaning, form, and discourse. The iconic gesture rate was positively correlated with both the three meaning-associated speech measures, including the number of word tokens (*r* = 0.452, *p* < 0.001), RTTR (*r* = 0.423, *p* < 0.001), and the speech rate (*r* = 0.407, *p* = 0.001), and the discourse-associated speech measure, i.e., the number of connectives per clause (*r* = 0.538, *p* < 0.001). However, none of the form-associated speech measures were correlated significantly with the iconic gesture rate.

**Table 10:** Correlations (Spearman's rho) between four types of co-speech gesture rate and speech measures.

	**Iconic rate**	**Metaphoric rate**	**Deictic rate**	**Beat rate**
Number of word tokens	0.452***	0.485***	0.225	0.333**
RTTR	0.423***	0.479***	0.207	0.231
Speech rate	0.407**	0.483***	0.265*	0.303*
Percentage of subordination	−0.236	−0.109	−0.222	−0.132
Mean length of clauses	0.097	0.154	0.230	−0.002
Percentage of error-free clauses	−0.138	0.312*	−0.062	−0.092
Number of connectives per clause	0.465***	0.212	0.354**	0.272*

* p < 0.05,

** p < 0.01,

*** p < 0.001.

Similar to the iconic gesture rate, the metaphoric gesture rate was correlated positively and significantly with all the meaning-associated speech measures. The Spearman's rho was 0.485 for the number of word tokens (*p* < 0.001), 0.479 for RTTR (*p* < 0.001), and 0.483 for the speech rate (*p* < 0.001). In addition, the metaphoric gesture rate was not significantly correlated with the percentage of subordination and the mean length of clauses, both form-associated measures. Unlike the iconic gesture rate, the metaphoric gesture rate was correlated positively with the percentage of error-free clauses (*r* = 0.312, *p* = 0.014), which is also a speech measure associated with language form, but had no significant correlation with the number of connectives per clause.

Significant correlations were fewer for the deictic gesture rate. It was correlated positively with the speech rate (*r* = 0.265, *p* = 0.039) and the number of connectives per clause (*r* = 0.354, *p* = 0.005); its correlation with the number of word tokens (*r* = 0.225, *p* = 0.082) was close to being significant and with RTTR nonsignificant. The correlations between the deictic gesture rate and form-associated speech measures were not significant.

The correlational relationships for beats were similar to, though weaker than, those involving iconics. The beat gesture rate was correlated positively with two of the meaning-associated speech measures, including the number of word tokens (*r* = 0.333, *p* = 0.009) and the speech rate (*r* = 0.303, *p* = 0.018), and the discourse-associated measure, i.e., the number of connectives per clause (*r* = 0.272, *p* = 0.034). Its correlation with RTTR trended toward significance (*r* = 0.231, *p* = 0.073). Again, beat gesturing had no significant correlation with any of the three form-associated speech measures.

### A summary: The associations between co-speech gestures and L2 speech performance

Positive associations with meaning-related L2 speech measures were observed for all the four types of co-speech gestures, and associations involving iconic gesturing and metaphoric gesturing were tighter. As shown in Table 11, gesturers of iconics performed better than non-gesturers of iconics in the three meaning-related measures, and this was also the case for metaphorics; in addition, both iconic and metaphoric rates were correlated positively with these speech measures. Deictic and beat gesturing had weaker associations with meaning-related speech measures. Though gesturers of deictics and gesturers of beats performed better in terms of the three meaning measures, the effect sizes were smaller compared with gesturers of iconics and metaphorics. Also, only one meaning-related speech measure (i.e., the speech rate) was correlated significantly with the deictic rate and two (i.e., the number of word tokens and the speech rate) with the beat rate in a positive way, and their effect sizes were again smaller compared with the iconic and metaphoric rates.

**Table 11:** Effect sizes and *p* values for associations between four types of co-speech gestures and meaning and discourse-related speech measures.

	**Iconics**	**Metaphorics**	**Deictics**	**Beats**
**Number of word tokens**				
Difference analysis	ω = 0.468, *p* < 0.001	*r* = 0.492, *p* < 0.001	ω = 0.268, *p* = 0.02	ω = 0.313, *p* = 0.008
Correlation analysis	*r* = 0.452, *p* < 0.001	*r* = 0.485, *p* < 0.001	*r* = 0.225, *p* = 0.082	*r* = 0.333, *p* = 0.009
**RTTR**				
Difference analysis	ω = 0.495, *p* < 0.001	*r* = 0.462, *p* < 0.001	ω = 0.311, *p* = 0.008	ω = 0.285, *p* = 0.014
Correlation analysis	*r* = 0.423, *p* < 0.001	*r* = 0.479, *p* < 0.001	*r* = 0.207, *p* = 0.11	*r* = 0.231, *p* = 0.073
**Speech rate**				
Difference analysis	ω = 0.449, *p* < 0.001	*r* = 0.485, *p* < 0.001	ω = 0.316, *p* = 0.007	ω = 0.345, *p* = 0.003
Correlation analysis	*r* = 0.407, *p* = 0.001	*r* = 0.483, *p* < 0.001	*r* = 0.265, *p* = 0.039	*r* = 0.303, *p* = 0.018
**Number of connectives per clause**				
Difference analysis	ω = 0.399, *p* < 0.001	*r* = 0.226, *p* = 0.077	ω = 0.346, *p* = 0.003	ω = 0.307, *p* = 0.009
Correlation analysis	*r* = 0.465, *p* < 0.001	*r* = 0.212, *p* = 0.101	*r* = 0.354, *p* = 0.005	*r* = 0.272, *p* = 0.034

Form-associated L2 speech measures were not significantly associated with any co-speech gestures except metaphorics. Compared with non-gesturers of metaphorics, gesturers of metaphorics produced a larger percentage of error-free clauses, and the metaphoric rate was significantly correlated with this measure of speech form.

For the discourse-associated L2 speech measure (i.e., the number of connectives per clause), the association with co-speech gestures was strongest for iconics, weaker for deictics and beats, and weakest for metaphorics, as shown in Table 11. Participants who used iconics employed more connectives per clause than participants who did not use iconics, and similar differences also existed for gesturers and non-gesturers of deictics and beats; furthermore, the iconic rate, deictic rate, and beat rate were significantly correlated with the number of connectives per clause. The use of iconics had the largest effect sizes followed by deictics and beats for both the difference analysis and the correlation analysis. Metaphoric gesture use had no significant association with the discourse measure.

## Discussion

This study explored the associations between co-speech gestures and speech performance for lower-intermediate L2 English language learners. We found that all the four types of co-speech gestures were more closely connected with meaning and discourse-associated L2 speech measures than with form-associated measures.

### Inter-gesture differences

One inter-gesture difference is that the associations between individual types of co-speech gestures and meaning-associated L2 speech measures were stronger for iconics and metaphorics, weaker for beats, and weakest for deictics. This was shown by the differences in effect sizes (see Table 11). For meaning-associated L2 speech measures, the effect sizes of the difference analysis for gesturers and non-gesturers of iconics and metaphorics were comparable, but the effect sizes were smaller for gesturers and non-gesturers of beats and deictics. In addition, in the correlational analysis, iconic and metaphoric rates had larger effect sizes, the beat rate had smaller effect sizes, and the deictic rate had the smallest effect sizes. Also, all three meaning-associated L2 speech measures were significantly correlated with iconic and metaphoric rates, two of them were significantly correlated with the beat rate, and only one with the deictic rate. This inter-gesture difference can be explained by findings from previous studies. First, as representational gestures, iconics and metaphorics have similar functions (Kita et al., 2017), and they can help gesturers conceptualize speech content (Alibali et al., 2000; Kita and Davies, 2009; Chu et al., 2014) and access words (Rauscher et al., 1996; Krauss et al., 2000; Frick-Horbury, 2002), which explains why they were conducive to meaning expression. Second, beats are also helpful in accessing words (Ravizza, 2003) and improving fluency (Vilà-Giménez and Prieto, 2020), but beats have a weaker association with speech content than representational gestures (McNeill, 1992). Third, deictics have the weakest association with speech performance measures and they tend to be used to compensate for weak speech proficiency (Nicoladis et al., 1999; Mayberry and Nicoladis, 2000; Nicoladis, 2002; Gan and Davison, 2011).

Another inter-gesture difference is that the association of the discourse-associated L2 speech measure, i.e., the number of connectives per clause, was the strongest with iconics, weaker with deictics and beats, and not significant with metaphorics (see Table 11). Whether or not L2 learners used iconics, deictics, and beats significantly influenced the use of connectives, and the effect sizes decreased from iconics, to deictics, and to beats. Correlation analysis displayed the same trend. The strongest association between iconics and connectives might be due to the function of iconics in conceptualizing speech content (Alibali et al., 2000; Kita and Davies, 2009; Chu et al., 2014), and the use of connectives is an important indicator of the quality of speech content on the discourse level. The reason why metaphorics, which are also representational in nature, bore little association with the use of connectives is unclear. It is possible that metaphorics were used so infrequently that no obvious association with connectives could be observed; it is also possible that by nature metaphorics are less likely to be used with connectives than with other speech measures. Both deictics and beats link relatively weakly to speech content (McNeill, 1992; Nicoladis et al., 1999; Mayberry and Nicoladis, 2000). Although previous studies have observed the co-occurrence of beats and connectives (McNeill, 1992; Dimitrova et al., 2016; Shattuck-Hufnagel and Ren, 2018), the association of beats with connective use was weaker than the association between iconics and connectives in this study, indicating that discourse cohesion is related more to the quality of speech content in general.

The third inter-gesture difference is the contrastive associations of individual types of co-speech gestures with the percentage of error-free clauses. The use of metaphoric gestures had a positive association with the percentage of error-free clauses, whereas the other three types of gestures bore negative, though insignificant, relations with it. This higher degree of accuracy for metaphorics was not due to shorter speech production and fewer opportunities to make language mistakes, since gesturers of metaphorics produced more word tokens than non-gesturers and the metaphoric gesture rate was correlated positively with the number of word tokens. Our results indicate that lower-intermediate L2 learners who use more metaphoric gestures also have a higher ability to monitor language mistakes. To our knowledge, no study has mentioned the function of metaphoric gestures in improving speech accuracy. In learning grammar, metaphoric gesturing is an important indicator to show L2 learners' learning process (Kimura and Kazik, 2017) and an effective interactional strategy to communicate with the lecturer about grammar learning (Matsumoto and Dobs, 2017). It is possible that our participants who benefited from gesturing in learning grammar also inherited such gesture use in monitoring language accuracy during speech production. Another possibility is that producing metaphoric gestures by schematizing abstract concepts saved the type of working memory resources that could be used for other tasks related to metaphoric thinking like monitoring grammar. Further studies are needed to investigate the mechanisms of this relationship.

### L2 speech dimensions associated with co-speech gesturing

Our results indicate that meaning and discourse-associated aspects of L2 speech measures are associated with co-speech gestures, whereas form-associated ones have weak associations with gestures. This is generally consistent with previous findings with regard to the functions of co-speech gestures, i.e., conceptualizing information and retrieving words (Rauscher et al., 1996; Alibali et al., 2000). Such functions can explain the associations between co-speech gesturing and speech length, lexical richness, speaking fluency, and discourse cohesion of L2 speech. Co-speech gestures' function of lowering cognitive load (Goldin-Meadow et al., 2001; Wagner et al., 2004; Ping and Goldin-Meadow, 2010; Cook et al., 2012; Chu et al., 2014) is also supported by our findings, but it seems that the working memory benefits brought by gesturing only contributed to improving the meaning expression for L2 learners, but not monitoring language form. Our participants were lower-intermediate level L2 learners for whom the cartoon-retelling task was challenging, and when they undertook the speech task, they were struggling to finish the task. In such cases, the cognitive resources created by using co-speech gestures might have been allocated primarily to conveying meaning. It is still unclear whether the particular measures of L2 speech associated with co-speech gesturing were influenced by language proficiency. It is possible that for more proficient language learners who find it unchallenging to retrieve words and express ideas, using co-speech gestures will benefit the speech performance measures related to form. Another possible explanation is that speakers who chose to gesture might have a greater desire to communicate ideas and thus spent more cognitive resources on meaning expression. More studies on the associations between gesturing, willingness to communicate, and speech performance are needed.

Our study demonstrates positive associations between connectives and gestures in a quantitative way. Using connectives is an important way to show semantic relations between textual constitutes (Halliday and Hasan, 1976). Their positive associations with co-speech gestures, namely iconics, beats, and deictics, show that co-speech gestures are helpful in not only meaning expression within clauses, but also meaning construction in the discourse.

The function of co-speech gesturing seems to resemble that of strategic pre-task planning in L2 speech production. Strategic planning makes learners prioritize meaning over form, such as producing speech with higher fluency and more diversified words (Yuan and Ellis, 2003; Sangarun, 2005; Li and Fu, 2018). Our participants were not allowed to prepare before the task, and co-speech gesturing was generally associated with measures related to speech meaning, such as the number of word tokens, RTTR that represents lexical richness, and the speech rate. This indicates that the cognitive resources released by using co-speech gestures might be similar to those provided by strategic planning. This possibility makes co-speech gestures a promising strategy when L2 learners face a challenging speech task with no preparation time. However, it is also found that, for advanced L2 learners, more representational and deictic gestures were produced when they were not allowed to do pre-task planning but more iconic ones were generated when they were allowed to do so (Lin, 2020), suggesting a more complex interaction between gesture, task planning, and language proficiency. To have more solid conclusions, future studies need to adopt experimental designs conductive to explore causal relationships between gesture and L2 speech performance, and take language proficiency and other task related factors into consideration.

### The associations between gesture and speech for L2 learners

Our study is a necessary complement to the current literature in that most relevant research focused on native and highly proficient bilingual speakers (Nicoladis et al., 2009; Smithson et al., 2011; Laurent and Nicoladis, 2015; Laurent et al., 2015). The associations between gesture use and speech performance reported in such studies were not consistent, which may have been caused by the ceiling effects resulted from participants' high language proficiency (Nicoladis et al., 2007). Our study explored the associations between co-speech gesturing and speech performance for lower-intermediate L2 learners, and we found some positive associations. With a lower language proficiency level, our participants were very likely to have faced a shortage of working memory resources and used gestures to facilitate speech production. It has been shown that the cognitive benefits of co-speech gestures are only obvious when participants face a high cognitive load (Marstaller and Burianová, 2013; Chu et al., 2014; Lewis et al., 2015). Co-speech gesturing produced by participants with lower language proficiency level in our study was not likely to be a reflection of speaking style (Nagpal et al., 2011), but more possibly a strategy to cope with the shortage of cognitive resources required by the speech task. Thus, the associations found in this study support the widely recognized close connection between gesture and speech. Our study can serve as a stepping stone to further research on the causal effect of co-speech gesturing on L2 speech performance. Such explorations have both pedagogical significance and practical value for L2 teachers and speakers.

## Conclusion

Based on the speech and gesture data elicited from a cartoon-retelling task completed by lower-intermediate L2 learners, this study found that all the four types of co-speech gestures were positively associated with meaning-related L2 speech measures, with the associations involving iconics and metaphorics stronger and that involving deictics and beats weaker; iconics, deictics and beats were also associated with L2 discourse cohesion in a positive way; and co-speech gestures had little association with form-associated L2 speech measures, except that metaphoric gestures were positively associated with the percentage of error-free clauses. The results show that all the four types of co-speech gestures tend to have a positive association with L2 speech meaning construction both within the clause and on the discourse level.

This study has several limitations. First, the interactions between speech, gestures, and other individual factors, such as personality, willingness to communicate, and working memory, were not taken into consideration. Second, participants were not required to gesture or not to; rather, they used or did not use gestures spontaneously. While this design eliminated possible influencing factors, it also made us lose the opportunity to explore the causal relationship between gesturing and speech. Third, since only L2 learners of one proficiency level were recruited as participants, whether the findings hold for speakers of other proficiency levels is unclear. Fourth, whether the findings apply to L2 learners with different language backgrounds requires further investigation. Lastly, our speech data were monologs instead of dialogues, which made it hard to explore interactional features of gesture and speech, such as pragmatic aspects of gestures (see Kendon, 2017 for more information). Future studies are needed to further explore the relationship between gesture and speech.

## Data availability statement

Researchers can contact the corresponding authors for access to de-identified data.

## Ethics statement

Ethical review and approval was not required for the study on human participants in accordance with the local legislation and institutional requirements. Written informed consent from the participants' legal guardian/next of kin was not required to participate in this study in accordance with the national legislation and the institutional requirements.

## Author contributions

SM wrote the first draft of the manuscript and GJ revised the draft. Both authors contributed to the conception and design of the study and data collection and analysis.

## Funding

This research was supported by the Fundamental Research Funds for the Central Universities in UIBE (Grant No. CXTD11-02). The fund was used to cover part of the salaries paid to research assistants and part of the processing fee.

## Conflict of interest

The authors declare that the research was conducted in the absence of any commercial or financial relationships that could be construed as a potential conflict of interest.

## Publisher's note

All claims expressed in this article are solely those of the authors and do not necessarily represent those of their affiliated organizations, or those of the publisher, the editors and the reviewers. Any product that may be evaluated in this article, or claim that may be made by its manufacturer, is not guaranteed or endorsed by the publisher.
